# Section on Ophthalmology

**Published:** 1899-01

**Authors:** Howard F. Hansell

**Affiliations:** Clerk of Section


### SECTION ON OPHTHALMOLOGY.

College of Physicians of Philadelphia. Meeting December 20, 1898. Dr. George C. Harlan Chairman, in the chair.

Dr. Charles A. Oliver detailed the history of a case of unequivocal *Reflex Urticaria Caused by Eye-strain.* The patient was a healthy, active woman, 47 years of age, who up to her 41st year had constantly suffered from a diffuse, unaccountable nettle-rash. Six years before she was seen by Dr. Oliver her physician found that a pair of complicated sphero-cylindrical bifocal lenses for constant use promptly, and without any suggestion or change of manner of life, prevented the urticarial attacks. A return of the eruption with visual disturbance, induced her four years later to seek a new cylindrical correction. Antimetropia, heterophoria, astigmatism, and presbyopia being corrected at this time, the cnidosic symptoms passed off, and have remained in abeyance ever since. Subjective and objective experiments of both motor and sensory type were made, both positively and negatively and locally and generally, until it was proven that a true relationship existed between the general vasomotor disturbance and the refractive and muscular anomalies.

Dr. John T. Carpenter, Jr., described a case of *Multiple Rupture at the Posterior Pole, with Associated Traumatic Lesions in the Iris and Lens*, in a boy of 15, the subject of convergent strabismus. Three years before, the right, the fixing eye, was struck by a potato thrown from a distance of 50 feet. The left eye was practically useless from high amblyopia and excessive convergence, and during the interval between the receipt of the injury to the other eye and the present, vision had remained equal to 1/60. In the right he had immediate loss of central vision, partially recovered to 6/24; pupil horizontally oval and widely dilated; paralysis of the sphincter of the upper half of the iris and trembling of the entire membrance; numerous "droplets" distributed in the anterior part of the lens-cortex and a linear opacity, vitreous shreds; papilla partly concealed by exudation; and finally 3 large tears in the choroid in the lower temporal and macular regions, partially including the nerve-head. Central vision was impossible. Dr. Carpenter called attention to the following points of interest: The associated lesions of the iris, lens, and fundust; the lack of improvement in vision of the amblyopic eye in 3 years; the number and character of the fundus lesions.

*Discussion.*—Dr. C. A. Oliver referred to several similar cases of his own and of Mr. Jonathan Hutchison. In one of the former a man was struck in the eye by a fist. The iris was ruptured, the sphincter split, the lens totally dislocated into the floor of the vitreous chamber, the choroid infiltrated with hemorrhages, the entire fundus hazy, and the disk atrophied.

Dr. H. F. Hansell explained the apparently contradictory history of Dr. Carpenter's case to that reported by Johnson, in which vision in the amblyopic eye had improved to the normal acuity after loss of the fixing eye, by noting that vision of the wounded eye was, in spite of the accident, better than the vision of the amblyopic eye; hence the latter was not used for fixation and the amblyopia remained without change.

Drs. G. E. de Schweinitz and C. A. Veasey presented the results of the *Bacteriological Examination of Forty six Cases of Conjunctivitis and Corneal Ulcer.* In general terms, in the mild types of conjunctivitis staphylococci were usually found; in the acute varieties, which were usually classified as contagious or epidemic, the pneumococci and the Koch-Weeks bacillus; in the membranous varieties either streptocci or Loffler's bacillus; and in the subacute forms of conjunctivitis, in one case at least, the diplo-bacillus of Morax and Axenfeld. In some cases unidentified bacteria were discovered, and in others the examinations were negative.

In the corneal ulcers of the superficial variety staphylococci and, in one instance, pneumococci were found; in the deep or sloughing ulcers without hypopyod, either streptococci or pneumococci; and in those with hypopyon, in one case a mixed infection (pneumococci and streptococci) and in the other a pure streptococcus infection.

Dr. de Schweinitz also described inoculation experiments on rabbits' eyes with pneumococcus cultures, in which he had failed to produce a typical serpent ulcer. Streptococcus injections and staphylococcus injections also failed to produce typical serpiginous ulcer.

The authors pointed out the value of bacteriological examinations, and gave illustrative cases where these examinations had influenced diagnosis, prognosis, and particularly therapeusis. While they realized the importance of bacteriological examination in all cases, they did not think our knowledge was sufficiently great as yet to make a bacteriological classification of the various types of conjunctivitis and ulcerative forms of keratitis to the exclusion of the clinical classification.

In the treatment of the membranous form, steptococcus conjunctivitis, Dr. Veasey relied principally upon frequent copious instillations of chlorate of potassium, gr. x–℥j, as suggested by Knapp. He was successful in removing the membrane with this remedy after unavailing use of mercury bichlorid., boric acid, formaldehyde, zinc, and other remedies, and considered its bactericidal action better than that of zinc solutions for this variety of conjunctivitis.

Dr. William M. Sweet read a paper on the *Diplo-bacillus of Chronic Catarrhal Conjunctivitis*, and reported the examination of 32 cases of conjunctival and supperative corneal disease, in

7 of which the germ was sound. Six of the patients had low-grade chronic conjunctival congestion, with moderate secretion, and 2 had corneal ulceration. In 1 case of phlyctenular conjunctivitis a few specimens of the germ were found. The life history of the organism agreed with the studies of its discoverer, Morax and Axenfeld, and with the investigations of Peters and Gifford, while the resistance of the disease to ordinary forms of treatment, and its prompt subsidence under solutions of zinc, were fully verified. In one case silver, tannin, boroglyceride, and boric acid were used as treatment for a period of five weeks, with no abatement of the symptoms. As to the germ having a distinct capsule, Dr. Sweet was disposed to agree with the findings of Gifford, although Morax, Axenfeld, and Peters state that no capsule exists.

*Discussion.*—Dr. de Schweinitz was inclined to believe in the existence of a bacillus capsule, although he admitted that it did not stain, and was difficult of demonstration. Dr. Oliver explained the exemption of some individuals and the selection of others on the basis of the difference in the character of the conjunctival sac and its secretions, as a soil .adapted to the development in some, and the death in others, of the bacilli.

Dr. W. C. Posey reported his experience with *De Weeker's Capsular Advancement Operation.* He has performed it in 33 cases, comprising exotropias of low degree and exophorias of high degree. The immediate gain was 50 degrees in many instances, but when measured two years later it was found that only 6 degrees of gain remained. Although the operation would seem, therefore, objectively to be of little advantage, subjectively, on the other hand, the results showed that the operation was justified, as the symptoms produced by the weakness of convergence were always ameliorated. The author found that the operation was without danger, as he had only slight reaction following its employment, nor had he noticed any disfigurement to the eye therefrom, as the fold in bulbar conjunctiva produced by the operation almost entirely disappeared at the end of a year.

Dr. Charles A. Oliver exhibited an apparatus designed to facilitate the use of Reid's ophthalmometer, which he claims furnishes distinct aid in accurately measuring the axis and degree of corneal astigmatism. The instrument is permanently held in an upright, adjustable contrivance of excellent workmanship and ingenious pattern.

HOWARD F. HANSELL,

Clerk of Section.

